# Extensive Natural Intraspecific Variation in Stoichiometric (C:N:P) Composition in Two Terrestrial Insect Species

**DOI:** 10.1673/031.008.2601

**Published:** 2008-03-27

**Authors:** S. M. Bertram, M. Bowen, M. Kyle, J. D. Schade

**Affiliations:** ^1^ Department of Biology, Carleton University, 1125 Colonel By Drive, Ottawa, Ontario, Canada K1S 5B6; ^2^ School of Life Sciences, Arizona State University, Tempe, AZ, 85287-4501; ^3^ Department of Biology, St. Olaf College, 1520 St. Olaf Avenue, Northfield, MN, 55057

**Keywords:** stoichiometry, carbon, nitrogen, phosphorus, cricket, *Gryllus*, weevil, *Sabinia*, essential elements

## Abstract

Heterotrophic organisms must obtain essential elements in sufficient quantities from their food. Because plants naturally exhibit extensive variation in their elemental content, it is important to quantify the within-species stoichiometric variation of consumers. If extensive stoichiometric variation exists, it may help explain consumer variation in life-history strategy and fitness. To date, however, research on stoichiometric variation has focused on *interspecific* differences and assumed minimal *intraspecific* differences. Here this assumption is tested. Natural variation is quantified in body stoichiometry of two terrestrial insects: the generalist field cricket, *Gryllus texensis* Cade and Otte (Orthoptera: Gryllidae) and a specialist curculionid weevil, *Sabinia setosa* (Le Conte) (Coleoptera: Curculionidae). Both species exhibited extensive intraspecific stoichiometric variation. Cricket body nitrogen content ranged from 8–12% and there was a four-fold difference in body phosphorus content, ranging from 0.32–1.27%. Body size explained half this stoichiometric variation, with larger individuals containing less nitrogen and phosphorus. Weevils exhibited an almost three-fold difference in body phosphorus content, ranging from 0.38–0.97%. Overall, the variation observed within each of these species is comparable to the variation previously observed across almost all terrestrial insect species.

## Introduction

Organisms that are incapable of making their own food must obtain their essential elements from their diet. However, plant nitrogen (N) and phosphorus (P) content is on average 6–16 times lower than it is in terrestrial insect herbivores (plants: ∼1.5%N, ∼0.05%P; insects: ∼9.3%N, ∼o.76%P, Elser et al. 2000; Pagan et al. 2002; Mattson 1980; Woods et al. 2004). This elemental mismatch between consumers and producers places inherent constraints on consumer ability to meet their nutritional requirements (Elser et al. 2000; Mattson 1980; Strong et al. 1984).

The ability of consumers to self-regulate their internal elemental content (stoichiometry) should critically influence how intensely diet quality affects herbivore fitness. If herbivores can maintain their stoichiometry regardless of diet quality they may reduce the fitness consequences of poor quality food. Research suggests, however, that consumers do not always maintain strict stoichiometric homeostasis. Instead, consumer body stoichiometry often tracks diet stoichiometry. For example, a laboratory experiment revealed that *Manduca sexta* caterpillars fed high phosphorus diets had significantly more phosphorus in their bodies than those fed low phosphorus diets (Perkins et al. 2004). Further, a field study revealed a tight association between soil phosphorus, mesquite phosphorus, and the body phosphorus content of *Sabina setosa*, a curculionid weevil that feeds on the mesquite. An increase in soil phosphorus was correlated with an increase in mesquite and weevil phosphorus (Schade et al. 2003). Overall, these results suggest diet stoichiometry may strongly influence consumer stoichiometry.

Both nitrogen and phosphorus are vitally important to insects. Nitrogen plays a fundamental role in protein production (Sterner and Elser 2002) and experimental manipulations reveal that consumer growth, reproduction, and survival are all strongly affected (Busch and Phelan 1999; Elser et al. 2003; Huberty and Denno 2006; Perkins et al. 2004). Phosphorus is also fundamentally tied to protein production through the synthesis of nucleic acids. While terrestrial insects are thought to be more limited by dietary nitrogen then by dietary phosphorus, the few studies that have examined the effects of dietary phosphorus have revealed that it can also affect growth rate and body size (Busch and Phelan 1999; Janssen 1994; Perkins et al. 2004), population density (Schade et al. 2003), reproduction (Popp et al. 1989), and survival (Clancy and King 1993). In a recent study examining the effects of both dietary nitrogen and phosphorus, Huberty and Denno (2006) found that elemental subsidies enhanced the body stoichiometry of the phytophagus planthopper, *Prokelisia marginata*. *P*. *marginata* raised on elementally enriched diets grew larger, developed more rapidly, and exhibited greater survival (Huberty & Denno 2006).

Because body stoichiometry appears to be intimately connected with insect growth rate, body size, reproduction, and survival, it is important to quantify the natural variation that exists among individuals in their body stoichiometry. Understanding the extent of this natural variation and the factors that affect it are necessary steps toward determining whether an ecological stoichiometric approach can help explain a fundamental evolutionary problem: the persistence of extensive variation in individual fitness in the face of strong directional selection.

To date, most studies examining insect stoichiometry have focused on taxonomic variation. Woods et al (2004) examined the body phosphorus content of 155 species of adult insects. Body phosphorus content was highly variable across taxa (range = 0.36% to 1.48%; mean = 0.79%), inversely correlated with insect size, and phylogenetically older orders tended to have more phosphorus (Woods et al. 2004). Only one individual was assayed in 98 of the 155 species examined and, when body phosphorus from two or more individuals were assayed, the results were averaged (Woods et al 2004). In a similar study, Fagan et al (2002) examined the body nitrogen content of 143 terrestrial insect species and revealed extensive variation across taxa (range = 5.7% to 13%; mean = 9.87%). Predators had significantly more nitrogen than herbivores; predator nitrogen was inversely correlated with body size, and phylogenetically older herbivorous lineages tended to contain more nitrogen (Fagan et al. 2002). All within-species estimates of body nitrogen content were averaged prior to analysis (Fagan et al 2002). Overall, both studies revealed a strong inverse relationship between body stoichiometry and body size across insect taxa. Both studies assumed that intraspecific stoichiometric variation was minimal.

Here the natural variation in body stoichiometry is quantified within two terrestrial insect species, the omnivorous Texas field cricket, *Gryllus texensis* Cade and Otte (Orthoptera: Gryllidae) and the specialist phloem feeding curculionid weevil, *Sabinia setosa* (Le Conte) (Coleoptera: Curculionidae). We chose the Texas field cricket because they exhibit extensive stoichiometric variation when reared in the laboratory on a high quality diet, suggesting that they might naturally display high intraspecific variation (Bertram et al. 2006). Because crickets are also highly variable in their body size (Bertram 2000), they also provide a natural intraspecific test of Fagan et al (2002) and Woods et al (2004) findings that an inverse relationship exists between body size and body stoichiometry across insect taxa. The curculionid weevil was chosen because its' body stoichiometry also appears highly variable; weevil body stoichiometry seems to track its' host plant stoichiometry which in turn tracks the natural variation in soil stoichiometry. Our overall goal was three-fold: (1) to quantify the natural variation within each of these two terrestrial insect species, (2) to determine whether body stoichiometry is inversely correlated to size within a species, and (3) to compare and contrast intraspecific variation to the previously described interspecific variation in terrestrial insects (Fagan et al. 2002; Woods et al. 2004).

## Materials and Methods

Adult male Texas field crickets (*G*. *texensis*) were captured (N = 78) in October, 2004 after being observed flying at the bright lights of a golf course driving range in Austin Texas. Austin crickets collected in this manner typically have a mean age of 11.1 ±2.6 (range = 7–18) days post final molt (Murray and Cade 1995). As part of a different experiment to quantify long distance mate attraction signals, each male was housed individually, provided with a water source, fed a uniform diet (4% N and 1.08% P; Harlan's Teklad Rodent Diet (W) 8604), and monitored acoustically for a one week period. Crickets were then placed in labeled plastic vials and frozen.

Crickets were dried at 60°C for ten days. Dried crickets were weighed to the nearest hundredth of a milligram using a Mettler MX5 electrobalance (http://us.mt.com/home). Each of the 78 crickets were then individually ground to a fine powder using a Spex Certiprep 8000D ball mill (www.spexcsp.com/). Body phosphorus content (percent of dry mass) was determined on several (mean = 3.9; range = 3–12 samples per individual) 1–2 mg powdered sub-samples of each cricket using the persulfate oxidation technique followed by analysis of orthophosphate using the acid molybdate technique (APHA 1992). Twelve of the 78 crickets were randomly selected for carbon and nitrogen analyses. Body carbon and nitrogen contents was analyzed using a Perkin-Elmer (www.perkinelmer.com) 2400 CHN elemental analyzer; a ∼4 mg powdered sub-sample was used. Cost limitations negated analyzing CHN of more than twelve individuals, or running more than one sample per cricket.

Adult curculionid weevils (*S*. *setosa*) were collected in May 2000, September 2000, and April 2001 from velvet mesquite trees, *Prosopis velutina* Woot. (Fabales: Fabaceae), found in three different habitats (riparian, desert, and woodland) in the Sonoran Desert near Phoenix, AZ. Insect nets were placed over the tree branch, the branch was pruned from the tree, and then shaken vigorously to dislodge the weevils. A sub-sample of weevils from each tree were dried (mean = 5.2). The phosphorus content of each individual was determined using the methodology described above. The entire individual was used in the phosphorus analysis because of the weevils' small body size (100 – 150 µg dry weight). It was therefore not possible to analyze CHN on these individuals. Population scale weevil data were originally published by Schade et al. (2003) in an examination of how soil phosphorus variation impacted mesquite and weevil phosphorus variation. In the present study the intra-specific phosphorus variation is compared and contrasted in weevils to the reported inter-specific phosphorus variation that exists across insects (Woods et al. 2004).

Statistical analyses were conducted using JMP software. The Shapiro-Wilk Goodness of Fit test was used to ensure data did not differ significantly from normal. Correlation analysis was used to examine the relationships between C, N, P, C:N, C:P, N:P and dry weight for the field-captured male crickets. Significance probabilities were adjusted to p<0.0165 to account for the 21 correlations (Dunn-Ŝidák method - Sokal and Rohlf 1995). We used an ANOVA to determine if field-captured weevils differ from field-captured crickets in their body phosphorus content. We analyzed the April 2001 seasonal weevil body phosphorus data separately from the phosphorus data collected in 2000 because body phosphorus content was significantly higher in 2001 than it was in the two 2000 collections (Schade et al. 2003). The May and September 2000 body phosphorus data did not differ significantly (Schade et al. 2003) so those two seasons were combined for subsequent analyses. The significance probability was adjusted to p<0.0253 to account for the 2 ANOVAs (Dunn-Ŝidák method). ANOVA was used to quantify potential influences of habitat (riparian, woodland, and desert) the weevils were collected from. The significance probability was adjusted to p<0.0253 to account for the 2 ANOVAs (Dunn-Ŝidák method).

**Table 1.**
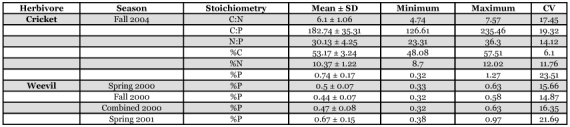
Organismal stoichiometric descriptive statistics for field-captured adult male Texas field crickets (*Gryllus texensis*) and field-captured adult curculionid weevil (*Sabinia setosa*).

## Results

Body stoichiometry of *G*. *texensis* exhibited extensive natural variation (Table 1). Body carbon content ranged from 48–57% total body mass, body nitrogen content ranged from 8–12%, and there was a four-fold difference in body phosphorus content, ranging from 0.32–1.27. The C:N and C:P ratios of adult male crickets both also exhibited an almost two-fold variation, ranging from 4.7 to 7.6, and 127 to 235, respectively. The N:P ratio ranged from 23 to 36. These results reveal that substantial stoichiometric variation exists within this one cricket species.

Carbon, nitrogen, and phosphorus content exhibited significant tradeoffs (Table 2). Cricket body carbon content was negatively correlated with nitrogen and phosphorus content. There was also a non-significant trend for nitrogen and phosphorus content to be positively correlated, suggesting that animals that are good at acquiring and retaining nitrogen are also good at acquiring and retaining phosphorus.

**Table 2.**
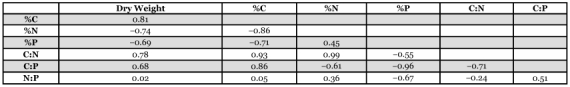
Correlations between dry weight and stoichiometric balance for field-captured crickets. Statistically significant correlations are in bold (statistical significance was reduced to P<0.0165 using the Dunn-Ŝidák method to account for 21 correlations). Stoichiometric ratios are atomic.

Heavier individuals contained more carbon and less nitrogen and phosphorus than lighter individuals (Table 2). Overall, 62% of variation in C, 51% of variation in N, and 47% of variation in phosphorus was explained by cricket dry weight (Regression: 96C: F = 19.02, p = 0.0014, R^2^adj = 0.62, df = 10,1; %N: F = 12.26, p = 0.0057, R^2^adj = 0.51, df = 10,1; %P: F = 68.58, p<0.0001, R^2^adj = 0.47, df = 76,1).

*S*. *setosa* also exhibited large variation in their body phosphorus content (Table 1). Weevils exhibited a two-fold difference in body phosphorus content in 2000, ranging from 0.32–0.63 %P. In 2001 the variation increased and individuals exhibited an almost three-fold difference in body phosphorus content, ranging from 0.38–0.97 %P. Body phosphorus content did not differ across the three habitats (riparian, desert, and woodland; ANOVA: 2000: F = 3.34, R^2^adj = 0.08, df = 49,2, P = 0.0435; 2001: F = 0.44, R^2^adj = -0.02, df = 53,2, P = 0.6470; the significance probability was adjusted to p<0.0253 to account for the multiple ANOVAs using the Dunn-Ŝidák method).

The weevils collected in 2001 did not differ from the crickets in their body phosphorus content (ANOVA: F = 5.09, R^2^adj = 0.03, df = 132,1, P = 0.0258; Table 1). However, the weevils collected in 2000 exhibited significantly lower body phosphorus contents than the crickets (ANOVA: F = 106.81, R^2^adj = 0.45, df = 128,1, P<0.0001; Table 1).

**Figure 1.**
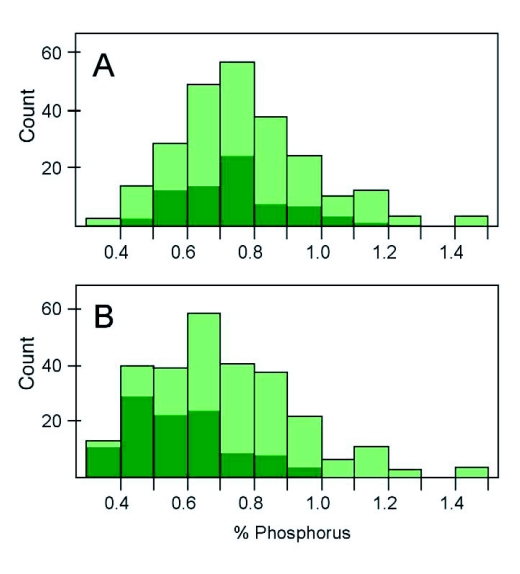
Comparing variation in total body phosphorus content within and across taxa. (A) The intraspecific variation in Texas field crickets (*Gryllus texensis*; dark bars) is compared to the interspecific variation (across insect taxa - light bars) in total body phosphorus content; (B) The intraspecific variation in Cuculinoid weevils (*Sabinia setosa*; dark bars) is compared to the interspecific variation (across insect taxa - light bars) in total body phosphorus content. Interspecific variation data are from Woods et al. (2004).

**Figure 2.**
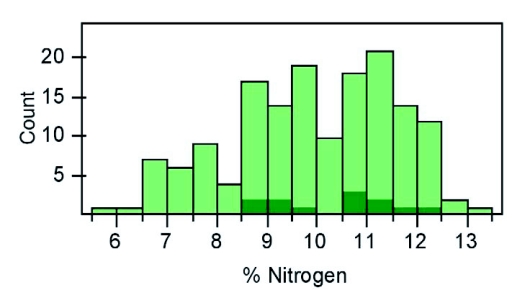
Comparing variation in total body nitrogen content within and across taxa. Intraspecific variation in Texas field crickets (*Gryllus texensis*; dark bars) is compared to the interspecific variation (across insect taxa - light bars) in total body nitrogen content. Interspecific variation data are from Fagan et al. (2004).

## Discussion

The cricket, *G*. *texensis*, exhibited substantial natural variation in their elemental stoichiometry. Crickets differed up to 20% in their body carbon content, up to 50% in their body N, and up to 400% in their body phosphorus content. Stoichiometric ratios were also highly variable with individuals differing up to 60% in their C:N, up to 85% in their C:P, and up to 60% in their N:P ratio. Overall, the intraspecific nitrogen and phosphorus variation in *G*. *texensis* was almost as high as the previously published interspecific variation estimates for terrestrial insect species (Figure 1a, 2). High intraspecific Stoichiometric variation was not limited to crickets: the specialist curculionid weevil, *S*. *setosa*, also exhibited substantial natural variation in body phosphorus (Figure 1b). These results suggest that high intraspecific Stoichiometric variation could be common across terrestrial insect taxa.

What might drive this extensive intraspecific variation in body stoichiometry? Approximately half the intraspecific Stoichiometric variation can be explained by individual differences in body size. Body stoichiometry is strongly and inversely correlated to body size. This inverse intraspecific relationship between body size and body stoichiometry is consistent with the inverse relationship observed across insect taxa; smaller species tend to have higher nitrogen and phosphorus contents than larger species (Fagan et al. 2002; Woods et al. 2004). Woods et al. (2004) and Fagan et al. (2002) provide a detailed discussion of what might drive this Stoichiometric allometry.

Differences in sex, stage, age, or wing morphology cannot explain the remaining observed intraspecific variation in our cricket samples because only flight capable adult males of similar age were used in these experiements. The crickets were collected while flying at lights. Macropterous (long winged and flight capable) males collected in this manner have a mean age of 11.1 ± 2.6 days post final moult (Murray and Cade 1995). The intraspecific variation in cricket stoichiometry can also not be explained by population differences because the crickets were all collected from one location over a one-week period. Variation in age, stage, or sex could, however, increase the intraspecific variation in body nitrogen and phosphorus beyond what was observed in this study. Larval *Drosophila melanogaster*, for example, exhibit substantial shifts in their nitrogen and phosphorus contents as they age. Young larvae tend to have 30% more nitrogen and 200% more phosphorus than larvae that are about to pupate (Watts et al. 2006).

Stoichiometric variation during developmental stages has been observed in the calanoid copepod (*Mixodiaptomus laciniatus*) (Carrillo et al. 2001). Nitrogen increases over 400% were measured during development from nauplii (mean ± SD = 1.4% ± 1.1) to copepodite (mean ± SD = 6.0% ± 2.1. Phosphorus increased across nauplii stages (stage Nil: mean ± SD = 0.75 ± 0.15; stage V: mean ± SD = 1.19 ± 0.17) but then declined across copepodite stages (stage CV: mean ± SD = 0.63 ± 0.07; (Carrillo et al. 2001). Likewise, female *D*. *melanogaster* contain significantly more phosphorus than males. Female *D*. *pseudoobscura*, for example, contain 74% more phosphorus than males; females: 1.14%P, males: 0.84%P (Markow et al. 1999). Thus, variation in age, stage, and/or sex could increase intraspecific Stoichiometric variation.

Variation among individuals in their body stoichiometry may result from differences in their ability to locate, consume, and retain sufficient essential elements from the diet. Terrestrial autotrophs display highly variable elemental contents that are typically poor in nitrogen and phosphorus (Elser et al. 2000). Autotrophic Stoichiometric variation might, therefore, partially explain the intraspecific variation in terrestrial insect stoichiometry. In support of this view, our research reveals that crickets reared in the laboratory on a constant diet exhibit less variation in their body nitrogen content (mean ± sd = 10.99 ± 0.98, range = 9.04 - 12.66, CV = 8.90, n = 55; (Bertram et al. 2006) than those that were captured as adults in the field after consuming presumably highly variable Stoichiometric diets (mean ± sd = 10.37 ± 1.22, range = 8.7 - 12.02, CV = 11.76, n = 12; this study). Similarly, the laboratory reared crickets exhibited substantially less variation in their body phosphorus content (mean ± sd = 0.67 ± 0.10, range = 0.44 – 0.87, CV = 14.95, n = 55; (Bertram et al. 2006) than those that were captured as adults in the field (mean ± sd = 0.74 ± 0.17, range = 0.32 – 1.27, CV = 23.5, n = 78; this study). Because intraspecific variation in body stoichiometry is high, future research on insects should examine how the ability to uptake and retain essential and limited elements influences intraspecific variation in fitness. Future research should also examine the relationship between body stoichiometry and the amount of energy stored, as variation among individuals in their energy stores might explain some of the observed intraspecific variation in nitrogen.

Studies examining stoichiometric variation across insect taxa indicate predators differ from herbivores in their body nitrogen content, and older insect lineages tend to contain more nitrogen and phosphorus than recently evolved liineages (Fagan et al. 2002; Woods et al. 2004). These studies were conducted under the assumption of minimal intraspecific stoichiometric variation. Our results suggest that the stoichiometric variation observed within crickets and weevils is almost as large as that previously observed across all insect taxa (Figure s1, 2). Future taxonomic studies should, therefore, take intraspecific stoichiometric variation into consideration.
